# *SIRT1* Polymorphism, Long-Term Survival and Glucose Tolerance in the General Population

**DOI:** 10.1371/journal.pone.0058636

**Published:** 2013-03-07

**Authors:** Sylwia M. Figarska, Judith M. Vonk, H. Marike Boezen

**Affiliations:** Department of Epidemiology, University Medical Center Groningen, University of Groningen, Groningen, The Netherlands; Sanjay Gandhi Medical Institute, India

## Abstract

Mutations that increase activity of Sir2 (silent information regulator 2) are associated with extended lifespan of yeast, fruit flies and worms. *SIRT1*, the human homolog of Sir2, that controls numerous physiological processes including the glucose metabolism, is considered a candidate gene for predicting variation in human lifespan. Whereas the role of Sir2 has been extensively investigated in model organisms, less is known about the relation between *SIRT1* and lifespan in humans. In the current study we included 1,390 subjects from a general population-based cohort with 18 years of follow-up to investigate associations between variation in single nucleotide polymorphisms (SNPs) in the *SIRT1* gene and human survival. Additionally in 535 male subjects with available data we investigated associations between *SIRT1* and glucose tolerance. Carriers of the minor allele of rs12778366 had a significantly reduced mortality risk compared to the wild types: Hazard Ratio 0.69 (95% CI 0.50 to 0.96; p = 0.025). The directions of the effect were the same in females and males, never and ever smokers and the effect was significantly protective in overweight/obese subjects. Carriers of the minor allele of SNP rs12778366 had better glucose tolerance indicated by 0.34 mmol/l lower glucose levels compared to wild type subjects (p = 0.03). This study shows that *SIRT1* affects human long-term survival and therefore may be an important factor in modulating lifespan not only in lower organisms, but also in humans.

## Introduction

In the ongoing quest to uncover factors that increase longevity, sirtuins have attracted scientific and public interest for the past decades [1]. Initially, overexpression of the silent information regulator Sir2, nicotinamide adenine dinucleotide (NAD^+^)-dependent histone deacetylase, has shown a beneficial effect on lifespan in budding yeast (*Saccharomyces cerevisae*) [2]. Subsequently, the experiments performed in worms (*Caenorhabditis elegans*) and flies (*Drosophila melanogaster*) [3], [4] confirmed favorable properties of Sir2, signifying the importance of sirtuins as longevity genes. Since a more recent report showed an absence of effects of Sir2 overexpression on lifespan in *C.elegans* and *Drosophila* [5], a debate about the role of sirtuins in lifespan prolongation has arisen [1]. Therefore more studies are needed to elucidate the impact of sirtuins on lifespan, especially in humans. Out of seven identified mammalian homologues, sirtuin 1 (*SIRT1*) is the most closely related to Sir2 [6]. *SIRT1* influences the activity of various transcription factors, including forkhead-box transcription factors (FOXOs), peroxisome proliferator-activated receptor γ (PPAR γ) and nuclear factor-κB (NF-κB) in target tissues resulting in enhanced gluconeogenesis and repressed glycolysis in the liver, reduction of adipogenesis in adipose tissue, and increased release of insulin in pancreatic beta cells [7]. SIRT1 controls adiponectin levels, inflammatory processes, gluconeogenesis, and levels of reactive oxygen species that together may lead to the development of insulin resistance [8]. Overexpression of SIRT1 or using SIRT1 activators improves glucose homeostasis and insulin sensitivity in mice [8]–[11]. Therefore partly the effect of SIRT1 on longevity may be exerted via its association with insulin signaling, which has been proven to extend lifespan by 18% in fat-specific insulin receptor knockout (FIRKO) mice [12]. Furthermore, SIRT1 is required for a normal response to caloric restriction that causes many changes in glucose metabolism and increases lifespan [13].

In humans, during the last years polymorphisms in *SIRT1* have been investigated in a context of metabolism and have been associated with BMI and risk of obesity [14]–[17], acute insulin response in Pima Indians [18], body fat and blood pressure in Japanese [19], basal energy expenditure and respiratory quotient [20], and with diabetes risk in interaction with prenatal exposure to famine [21]. The few studies that investigated SNPs in *SIRT1* in relation to human lifespan or mortality did not find any associations [22]–[25].

Given the fact that near 30% of the individual variance in life expectancy is genetically determined [26] and the specific genetic determinants of human lifespan still remain largely unknown, *SIRT1*, as a metabolic master switch [7], may be considered a candidate gene for predicting variation in human lifespan. The Vlagtwedde/Vlaardingen cohort offers the unique opportunity to investigate the role of *SIRT1* in long-term survival, because subjects included in the current study were followed up for 18 years. Since *SIRT1* modulates a range of cellular processes involved in maintaining glucose homeostasis [27], we additionally investigated *SIRT1* polymorphisms and glucose tolerance.

## Methods

### Ethics Statement

The study protocol was approved by the local university medical hospital ethics committee, University of Groningen, University Medical Center Groningen, The Netherlands and all participants gave their written informed consent. In 1984, the Committee on Human Subjects in Research of the University of Groningen reviewed the study and affirmed the safety of the protocol and study design.

### Study population

We studied 1,390 subjects of the Vlagtwedde/Vlaardingen cohort participating in the last survey in 1989/1990 [28]. This general population-based cohort of white individuals of Dutch descent started in 1965 and has been followed for 25 years. The main focus of the study was on respiratory health. Surveys (median number of 7 per subject, range 1–8) were performed every 3 years, during which information was collected on smoking status, age, sex and respiratory symptoms by the Dutch version of the British Medical Council standardized questionnaire, BMI was determined, spirometry was performed and and the number of eosinophils in peripheral blood was measured. The vital status of all participants in the study on December 31, 2008 was assessed. Causes of death were coded according to the International Classification of Diseases (ICD) and obtained from the Statistics Netherlands (The Hague). In order to avoid bias and provide true associations, the external causes of death (i.e. suicides, homicides, traffic accidents etc.) were excluded from the analyses, (ICD-9: codes≥800 and in ICD-10: codes ≥S00).

### Blood samples

In 1989/1990 neutrophil depots from peripheral blood samples were collected and stored at –20°C. In 2003–2004 DNA was extracted from these samples with a QIAamp DNA blood mini kit (Qiagen, Hilden, Germany) and checked for purity and concentration with a NanoDrop ND-1000 UV–Vis spectrophotometer (NanoDrop Technologies, Wilmington, DE) [28].

### SNP Selection and Genotyping

Four SNPs (rs12778366, rs10823108, rs7069102 and rs2273773), that tag all 21 SNPs in *SIRT1* and its 5 kb up-/downstream region with r^2^>0.8 and Minor Allele Frequency>5% (based on the HapMap release 23a/March 2008) were genotyped by K-Bioscience Ltd (UK) [29]. Since rs10823108 and rs2273773 were in complete linkage disequilibrium (r^2^ = 1.0, Figure S1) in our study population, only rs2273773 was analyzed.

### Oral glucose tolerance test

In 1970/1972, 1973 and 1976 male subjects underwent the oral glucose tolerance test (OGTT). They were given a drink of 100 g glucose solution, and blood glucose was measured two hours later.

### Statistical Analysis

Descriptive analyses of the subject characteristics were performed using χ^2^ tests for categorical variables and Mann-Whitney U test for continuous variables (i.e. packyears in ever smokers and age). The genotype frequencies were tested for Hardy-Weinberg Equilibrium (HWE) by χ^2^ analysis. Differences in genotype distribution between dead and alive subjects were tested using χ^2^ tests. SNP rs10823108 was tested in a general genetic model. Due to the low frequency of individuals being homozygous for the minor allele for rs12778366 (n = 14) and rs2273773 (n = 9) heterozygotes and homozygotes for the minor allele were analyzed in a one group. Cox proportional hazards regression models adjusted for gender, age and packyears of smoking (all at the survey in 1989/1990) were used to evaluate the association between SNPs and all-cause mortality. Time was defined from the examination in 1989/1990 until death, end of follow-up in 2008 or last registration if subjects were lost to follow-up. Survival curves are depicted based on these Cox models. Stratified analyses according to gender, smoking habits (never smokers vs ever smokers), BMI and age (dichotomized based on a median age at visit in 1989/1990, i.e. 52 yrs) were performed. Subjects with BMI ≥25 kg/m^2^ were categorized into the overweight/obese group according to World Health Organization (WHO) criteria.

A linear regression model adjusted for age at the measurement was used to evaluate the associations between SNPs in *SIRT1* and glucose tolerance.

P values <0.05 were considered statistically significant (tested 2-sided). All statistical analyses were performed using SPSS version 18.0 for Windows.

## Results

Subjects with genetic data available and participating in the last survey in 1989/1990 were included in this study (n = 1,390, see Table 1). After 18 years of follow-up, 78.2% (n = 1,087) of the cohort was still alive and 284 deaths (20.4%) were recorded. Out of all deaths, 14 (4.9%) occurred due to external causes and these were excluded form the analyses. Of the participants who had died 207 (76.7%) were ever smokers and these had significantly higher numbers of packyears compared to participants still alive. It is important to note that our study had an excellent follow-up rate, since only 19 subjects (1.4%) could not be traced back.

**Table 1:** Characteristics of participants at visit 1989/1990 by vital status on Dec 31^st^, 2008.

Status on 31-12-2008	Alive	Dead	p value
Number (%)	1087 (78.2)	270 (19.4)	
Males, n (%)	525 (48.3)	166 (61.5)	0.000
Age, median (range)	49.4 (36.0 to 72.6)	61.8 (37.3 to 79.1)	0.000
Ever smokers, n (%)	711 (65.4)	207 (76.7)	0.000
Packyears in ever smokers, median (range)	17.2 (0.1 to 117.1)	26.0 (0.6 to 262.2)	0.000
BMI			
Normal weight, n (%)	284 (26.2)	65 (24.2)	
Overweight, n (%)	540 (49.8)	139 (51.6)	
Obese, n (%)	261 (24.0)	65 (24.2)	0.781

The 3 tested SNPs (rs12778366, rs7069102 and rs2273773) were in Hardy-Weinberg equilibrium. Among subjects who died, 83% had the wild type genotype of rs12778366 which is significantly higher (p = 0.015) than the 76% in the subjects who were still alive.

**Figure 1:**
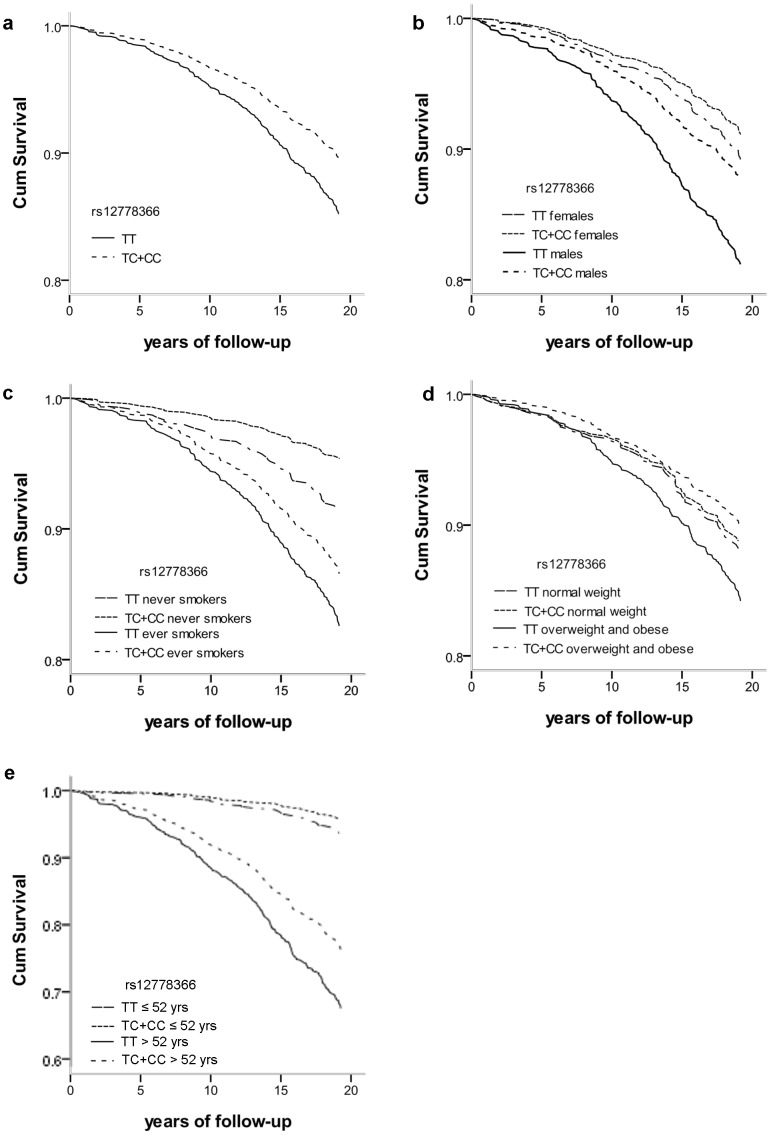
Survival curves for all-cause mortality according to SNP rs12778366. **a**. all subjects; **b**. stratified according to gender; **c**. stratified according to smoking habits; **d**. stratified according to BMI; **e**. stratified according to age (median age at visit in 1989/1900). *the Y axis scale in a figure 1e differs from Y axis scales in other figures.

Cox regression showed that carriers of the minor allele of rs12778366 had a significantly reduced risk of mortality compared to wild types: HR 0.69 (95% CI: 0.50 to 0.96; p = 0.025; see Table 2). Survival curves according to genotypes of rs12778366 clearly show the difference in mortality risk (Figure 1a). The same directions of the effect of rs12778366 were observed within groups that have different mortality risks, i.e. females (HR = 0.82 (0.50−1.35)) and males (HR = 0.63 (0.41−0.96)), never smokers (HR = 0.53 (0.25−1.11)) and ever smokers (HR = 0.75 (0.52−1.08)), younger subjects (age ≤ 52 yrs) (HR = 0.68 (0.30−1.54)) and older subjects (age > 52 yrs) (HR = 0.69 (0.48−0.98)), (Table 3 and Figure 1 b–c, e). Remarkably, the protective effect of rs12778366 was observed in overweight/obese subjects (HR = 0.62 (0.43−0.91)) but not in subjects with normal weight (HR = 0.95 (0.50−1.80). The survival curves clearly show that overweight/obese minor allele carriers of rs12778366 had survival comparable to subjects with normal weight while overweight/obese subjects with the wildtype genotype had an increased mortality risk (Figure 1d). The 2 other SNPs did not show significant associations between genotypes and mortality risk (Table 2).

**Table 2:** Distribution of genotypes and hazard ratio (HR) for all-cause mortality.

SNP	Genotype	Alive n = 1,087	All-cause mortality n = 270	p value**	HR (95% CI)***	p value
rs12778366*	TT	798 (76.0)	220 (83.0)		1	
	TC+CC	252 (24.0)	45 (17.0)	0.015	0.69 (0.50−0.96)	0.025
rs7069102	GG	449 (43.6)	124 (48.8)		1	
	GC	458 (44.5)	103 (40.6)		0.85 (0.65−1.11)	0.234
	CC	123 (11.9)	27 (10.6)	0.323	0.90 (0.59−1.37)	0.628
rs2273773*	TT	897 (81.1)	164 (76.6)		1	
	TC+CC	209 (18.9)	50 (23.4)	0.132	1.26 (0.92−1.73)	0.155

* Due to the low frequency of individuals being homozygous for the minor allele heterozygotes and homozygotes variants were combined

** Differences in genotype distribution between alive subjects and those who died (excluding external causes of death) tested with χ^2^ test

*** Cox regression adjusted for age, gender and packyears at visit in 1989/90

**Table 3:** HR for all-cause mortality for rs12778366 TC+CC genotypes in stratified analysis.

Stratification	HR (95% CI)**	p value
a) gender		
Females, n* = 653	0.82 (0.50−1.35)	0.424
Males, n = 680	0.63 (0.41−0.96)	0.032
b) smoking habits		
Never smokers, n = 424	0.53 (0.25−1.11)	0.092
Ever smokers, n = 909	0.75 (0.52−1.08)	0.125
c) BMI		
Normal weight, n = 344	0.95 (0.50−1.80)	0.870
Overweight and obese, n = 987	0.62 (0.43−0.91)	0.014
d) age		
≤ 52 yrs, n = 670	0.68 (0.30−1.54)	0.350
> 52 yrs, n = 663	0.69 (0.48−0.98)	0.040

* n = number of all subjects included in the analysis (excluding those who died due to external causes)

** rs12778366 TT genotype as a reference

We analyzed the available data from 535 male subjects from the current study (aged at the measurement 18–61 years) who underwent the glucose tolerance test (OGTT). We found that the minor allele carriers of SNP rs12778366 had better glucose tolerance, since they had a 0.34 mmol/l lower glucose levels compared to wild type subjects (p = 0.03, see Table 4). When this association was further investigated in subjects with normal weight (n = 249) and overweight/obese subjects (n = 284) separately, we found significantly better glucose tolerance in overweight/obese carriers of the minor allele of rs12778366 (i.e. 0.60 mmol/l lower glucose levels (p = 0.01)) compared to overweight/obese wild type subjects. In subjects with normal weight the same direction was observed (0.18 mmol/l lower glucose levels), but the effect was not significant (p = 0.50).

**Table 4:** Glucose levels (mmol/l) measured in males after the oral glucose tolerance test (OGGT).

SNP	Genotype	n	Mean (SD)	B* (mmol/l)	SE	p value
rs12778366	TT	414	6.33 (1.55)			
	TC+CC	119	5.99 (1.22)	−0.34	0.16	0.030
rs7069102	GG	229	6.33 (1.59)			
	GC	232	6.02 (1.43)	−0.12	0.14	0.401
	CC	59	6.32 (1.44)	0.02	0.22	0.931
rs2273773	TT	446	6.27 (1.51)			
	TC+CC	89	6.22 (1.44)	−0.04	0.17	0.830

* Regression coefficient (B), its standard error (SE) and p value obtained with linear regression analysis adjusted for age at the measurement.

## Discussion

We found a 30% reduced mortality risk among minor allele carriers of SNP rs12778366 in *SIRT1*, during a 18 years follow-up study in the general population. Therefore, *SIRT1* appears to be an important candidate gene explaining individual differences in human lifespan. There is evidence linking overexpression of Sir2 to extended lifespan in yeast, worms and flies [2]–[4]. Despite the established role of mammalian *SIRT1* in metabolism, genome stability and stress response [30], [31], polymorphisms in *SIRT1* were not associated with exceptional human longevity in a cross-sectional case-control study [22], [24] nor with all-cause mortality in a general population-based cohort [25] and in a group of over 85 years who were followed up until they died [23]. Whereas the last study included only pre-selected old subjects the advantage of our current study is that we did not use a selection criteria based on age, but investigated the whole population in a longitudinal manner. Actually, up till now, only one study in humans showed associations between *SIRT1* variants and healthy aging, what the authors defined as being healthy (i.e. normal brain function and verbal fluency test; laboratory findings for hemogram, peripheral smear, urine, electrocytes, chest X-ray, kidney, pulmonary function, echocardiography and ECG were normal) at the age 60 or higher [32]. In the light of recent uncertainty about the role of sirtuins in longevity [1] our results importantly provide new evidence in favor of a role of the gene in longevity.

We have shown an association between the *SIRT1* gene and long-term survival among minor allele carriers of SNP rs12778366 in *SIRT1* in the total population. Furthermore, the directions of the effect did not change in stratified analyses according to gender, smoking habits and age. Interestingly, stratification according to BMI showed the protective effect of rs1277836 only in overweight/obese subjects. Taking into account the increased mortality per se in obese subjects this finding may shed a new light on obesity-related burdens.

One of the physiological pathways through which *SIRT1* may affect longevity might be glucose homeostasis. Indications suggesting a role of this pathway were backed up by evidence that minor allele carriers of SNP rs12778366 had better glucose tolerance as determined by the oral glucose tolerance test. Interestingly, stratified analysis according to BMI showed that the effect was more pronounced in overweight/obese subjects, whereas in subjects with normal weight only the direction of the effect remained the same, but was not significant. In this light the better glucose tolerance in overweight/obese minor allele carriers of SNP rs12778366 could be considered a condition leading to better survival in this group. This additional result emphasizes the relevance of rs12778366 and indicates its possible use as a screening tool for clinical purposes.

Previous studies indicate that transgenic mice that overexpress *SIRT1* appear to have beneficial phenotypes that may be relevant in human health, including better glucose tolerance [33], [34]. In contrast to the positive effects of increased *SIRT1* activity, *SIRT1* deficiency impairs metabolism [35]. Therefore, we hypothesize that variants in rs12778366 may lead to overexpression of the protein, especially since this SNP is located in Transcription Factor Binding Site (TFBS). Although rs12778366 is not associated with the SIRT1 protein expression in adipose tissue, lymphoblastoid cell lines and skin (Genevar (GENe Expression VARiation) database) [36], this does not rule out a possible effect of the SNP on protein expression in other tissues i.e. in the lung, liver or heart, given the broad *SIRT1* expression in humans.

### Strengths and limitations

The major strength of the current study is the longitudinal design. We were able to follow participants for 18 years, which provided a wide time window for evaluating survival in the cohort. A strength of our study is also the number of subjects (n = 1,390), sampled from the general population. Additionally, the high follow-up rate is a major strength of the study, since 98.6% of the included subjects could be traced back. A limitation of our study is the limited data on metabolic profile, because the oral glucose tolerance test was performed only in male subjects and was not accompanied by insulin measurements. Furthermore, the study population consisted of white individuals of Dutch descent which limits extrapolation to other ethnic groups.

In summary, this is the first study showing that *SIRT1* plays a role in human lifespan in a non-selected general population cohort. The importance of *SIRT1* is supported by the association of its polymorphism with long-term survival in the general population. Furthermore, linking *SIRT1* polymorphisms to improved glucose tolerance stresses the impact of *SIRT1* on metabolism in humans and identifies *SIRT1* as a possible candidate for therapeutic purposes.

## Supporting Information

Figure S1 ***SIRT1*** **linkage disequilibrium plot (100·r^2^) in the Vlagtwedde/Vlaardingen cohort.**
(TIF)

## References

[1] Accili D, de Cabo R, Sinclair DA (2011) An unSIRTain role in longevity. Nat Med 17: 1350–1351. 22064411 10.1038/nm1111-1350

[2] Kaeberlein M, McVey M, Guarente L (1999) The SIR2/3/4 complex and SIR2 alone promote longevity in Saccharomyces cerevisiae by two different mechanisms. Genes Dev 13: 2570–2580. 10521401 10.1101/gad.13.19.2570 PMC317077

[3] Rogina B, Helfand SL (2004) Sir2 mediates longevity in the fly through a pathway related to calorie restriction. Proc Natl Acad Sci U S A 101: 15998–6003. 15520384 10.1073/pnas.0404184101 PMC528752

[4] Tissenbaum HA, Guarente L (2001) Increased dosage of a sir-2 gene extends lifespan in Caenorhabditis elegans. Nature 410: 227–230. 11242085 10.1038/35065638

[5] Burnett C, Valentini S, Cabreiro F, Goss M, Somogyvari M, et al (2011) Absence of effects of Sir2 overexpression on lifespan in C. elegans and Drosophila. Nature 477: 482–485. 21938067 10.1038/nature10296 PMC3188402

[6] Frye RA (1999) Characterization of five human cDNAs with homology to the yeast SIR2 gene: Sir2-like proteins (sirtuins) metabolize NAD and may have protein ADP-ribosyltransferase activity. Biochem Biophys Res Commun 260: 273–279. 10381378 10.1006/bbrc.1999.0897

[7] Leibiger IB, Berggren PO (2006) Sirt1: a metabolic master switch that modulates lifespan. Nat Med 12: 34–36. 16397557 10.1038/nm0106-34

[8] Liang F, Kume S, Koya D (2009) SIRT1 and insulin resistance. Nat Rev Endocrinol 5: 367–373. 19455179 10.1038/nrendo.2009.101

[9] Li Y, Xu S, Giles A, Nakamura K, Lee JW, et al (2011) Hepatic overexpression of SIRT1 in mice attenuates endoplasmic reticulum stress and insulin resistance in the liver. FASEB J 25: 1664–1679. 21321189 10.1096/fj.10-173492 PMC3079300

[10] Feige JN, Lagouge M, Canto C, Strehle A, Houten SM, et al (2008) Specific SIRT1 activation mimics low energy levels and protects against diet-induced metabolic disorders by enhancing fat oxidation. Cell Metab 8: 347–358. 19046567 10.1016/j.cmet.2008.08.017

[11] Lagouge M, Argmann C, Gerhart-Hines Z, Meziane H, Lerin C, et al (2006) Resveratrol improves mitochondrial function and protects against metabolic disease by activating SIRT1 and PGC-1alpha. Cell 127: 1109–1122. 17112576 10.1016/j.cell.2006.11.013

[12] Bluher M, Kahn BB, Kahn CR (2003) Extended longevity in mice lacking the insulin receptor in adipose tissue. Science 299: 572–574. 12543978 10.1126/science.1078223

[13] Boily G, Seifert EL, Bevilacqua L, He XH, Sabourin G, et al (2008) SirT1 regulates energy metabolism and response to caloric restriction in mice. PLoS One 3: e1759. 18335035 10.1371/journal.pone.0001759 PMC2258149

[14] Clark SJ, Falchi M, Olsson B, Jacobson P, Cauchi S, et al (2012) Association of Sirtuin 1 (SIRT1) Gene SNPs and Transcript Expression Levels With Severe Obesity. Obesity (Silver Spring) 20: 178–185. 21760635 10.1038/oby.2011.200 PMC3760128

[15] Peeters AV, Beckers S, Verrijken A, Mertens I, Roevens P, et al (2008) Association of SIRT1 gene variation with visceral obesity. Hum Genet 124: 431–436. 18820948 10.1007/s00439-008-0567-8

[16] Zheng J, Chen LL, Xiao F, Hu X, Deng X, et al (2012) Three single nucleotide variants of the SIRT1 gene are associated with overweight in a Chinese population: a case control study. Endocr J 59: 229–237. 22230810 10.1507/endocrj.ej11-0234

[17] Zillikens MC, van Meurs JB, Rivadeneira F, Amin N, Hofman A, et al (2009) SIRT1 genetic variation is related to BMI and risk of obesity. Diabetes 58: 2828–2834. 19741164 10.2337/db09-0536 PMC2780870

[18] Dong Y, Guo T, Traurig M, Mason CC, Kobes S, et al (2011) SIRT1 is associated with a decrease in acute insulin secretion and a sex specific increase in risk for type 2 diabetes in Pima Indians. Mol Genet Metab 104: 661–665. 21871827 10.1016/j.ymgme.2011.08.001 PMC3224181

[19] Shimoyama Y, Suzuki K, Hamajima N, Niwa T (2011) Sirtuin 1 gene polymorphisms are associated with body fat and blood pressure in Japanese. Transl Res 157: 339–347. 21575918 10.1016/j.trsl.2011.02.004

[20] Weyrich P, Machicao F, Reinhardt J, Machann J, Schick F, et al (2008) SIRT1 genetic variants associate with the metabolic response of Caucasians to a controlled lifestyle intervention--the TULIP Study. BMC Med Genet 9: 100. 19014491 10.1186/1471-2350-9-100 PMC2626584

[21] Botden IP, Zillikens MC, de RooijSR, Langendonk JG, Danser AH, et al (2012) Variants in the SIRT1 gene may affect diabetes risk in interaction with prenatal exposure to famine. Diabetes Care 35: 424–426. 22228742 10.2337/dc11-1203 PMC3263901

[22] Flachsbart F, Croucher PJ, Nikolaus S, Hampe J, Cordes C, et al (2006) Sirtuin 1 (SIRT1) sequence variation is not associated with exceptional human longevity. Exp Gerontol 41: 98–102. 16257164 10.1016/j.exger.2005.09.008

[23] Kuningas M, Putters M, Westendorp RG, Slagboom PE, van Heemst D (2007) SIRT1 gene, age-related diseases, and mortality: the Leiden 85-plus study. J Gerontol A Biol Sci Med Sci 62: 960–965. 17895433 10.1093/gerona/62.9.960

[24] Willcox BJ, Donlon TA, He Q, Chen R, Grove JS, et al (2008) FOXO3A genotype is strongly associated with human longevity. Proc Natl Acad Sci U S A 105: 13987–13992. 18765803 10.1073/pnas.0801030105 PMC2544566

[25] Zillikens MC, van Meurs JB, Sijbrands EJ, Rivadeneira F, Dehghan A, et al (2009) SIRT1 genetic variation and mortality in type 2 diabetes: interaction with smoking and dietary niacin. Free Radic Biol Med 46: 836–841. 19167483 10.1016/j.freeradbiomed.2008.12.022

[26] Herskind AM, McGue M, Holm NV, Sorensen TI, Harvald B, et al (1996) The heritability of human longevity: a population-based study of 2872 Danish twin pairs born 1870–1900. Hum Genet 97: 319–323. 8786073 10.1007/BF02185763

[27] Houtkooper RH, Pirinen E, Auwerx J (2012) Sirtuins as regulators of metabolism and healthspan. Nat Rev Mol Cell Biol 13: 225–238. 22395773 10.1038/nrm3293 PMC4872805

[28] van Diemen CC, Postma DS, Vonk JM, Bruinenberg M, Schouten JP, et al (2005) A disintegrin and metalloprotease 33 polymorphisms and lung function decline in the general population. Am J Respir Crit Care Med 172: 329–333. 15879414 10.1164/rccm.200411-1486OC

[29] Siedlinski M, Boer JM, Smit HA, Postma DS, Boezen HM (2012) Dietary factors and lung function in the general population: wine and resveratrol intake. Eur Respir 39: 385–391. 10.1183/09031936.00184110 21852339

[30] Guarente L (2011) Franklin H. Epstein Lecture: Sirtuins, aging, and medicine. N Engl J Med 364: 2235–2244. 21651395 10.1056/NEJMra1100831

[31] Chua KF, Mostoslavsky R, Lombard DB, Pang WW, Saito S, et al (2005) Mammalian SIRT1 limits replicative life span in response to chronic genotoxic stress. Cell Metab 2: 67–76. 16054100 10.1016/j.cmet.2005.06.007

[32] Zhang WG, Bai XJ, Chen XM (2010) SIRT1 variants are associated with aging in a healthy Han Chinese population. Clin Chim Acta 411: 1679–1683. 20633545 10.1016/j.cca.2010.06.030

[33] Bordone L, Cohen D, Robinson A, Motta MC, van Veen E, et al (2007) SIRT1 transgenic mice show phenotypes resembling calorie restriction. Aging Cell 6: 759–767. 17877786 10.1111/j.1474-9726.2007.00335.x

[34] Moynihan KA, Grimm AA, Plueger MM, Bernal-Mizrachi E, Ford E, et al (2005) Increased dosage of mammalian Sir2 in pancreatic beta cells enhances glucose-stimulated insulin secretion in mice. Cell Metab 2: 105–117. 16098828 10.1016/j.cmet.2005.07.001

[35] Baur JA, Ungvari Z, Minor RK, Le Couteur DG, de Cabo R (2012) Are sirtuins viable targets for improving healthspan and lifespan? Nat Rev Drug Discov 11: 443–461. 22653216 10.1038/nrd3738 PMC4684642

[36] Yang TP, Beazley C, Montgomery SB, Dimas AS, Gutierrez-Arcelus M, et al (2010) Genevar: a database and Java application for the analysis and visualization of SNP-gene associations in eQTL studies. Bioinformatics 26: 2474–2476. 20702402 10.1093/bioinformatics/btq452 PMC2944204

