# Supplementary figures and images for: SIRT1 Polymorphism, Long-Term Survival and Glucose Tolerance in the General Population
Source: PLoS One. 2013 Mar 7;8(3):e58636. doi: 10.1371/journal.pone.0058636 (PMC3591365; doi:10.1371/journal.pone.0058636)

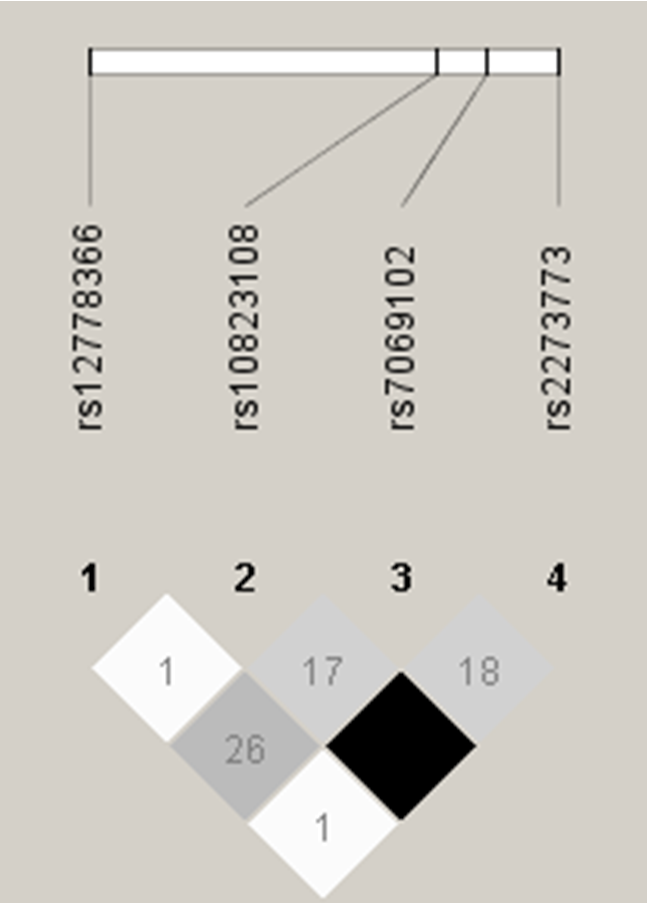

Supplement: Figure S1 — SIRT1 linkage disequilibrium plot (100·r2) in the Vlagtwedde/Vlaardingen cohort. (TIF) [file pone.0058636.s001.tif]
